# A Rent Subsidy and Identity Capital Intervention for Youth Exiting Homelessness: Protocol for the Transitioning Youth Out of Homelessness 2.0 Pilot Randomized Controlled Trial

**DOI:** 10.2196/66210

**Published:** 2025-04-25

**Authors:** Naomi S Thulien, Rowen K Stark, Alexandra Amiri, Alex Abramovich, Alex Akdikmen, Alexandra Carasco, Mardi Daley, Bernice Downey, Oluwapelumi (Pukky) Fambegbe, Tyler Frederick, Stephen W Hwang, Nicole Kozloff, Amanda Noble, Cheryl Pedersen, Marsha Rampersaud, Ruth Rodney, Tadios Tibebu, Rosane Nisenbaum

**Affiliations:** 1 MAP Centre for Urban Health Solutions Li Ka Shing Knowledge Institute of St Michael's Hospital Unity Health Toronto Toronto, ON Canada; 2 Social & Behavioral Health Sciences Division Dalla Lana School of Public Health University of Toronto Toronto, ON Canada; 3 Centre for Critical Qualitative Health Research University of Toronto Toronto, ON Canada; 4 Harvard T.H. Chan School of Public Health Harvard University Boston, MA United States; 5 Institute for Mental Health Policy Research Centre for Addiction and Mental Health Toronto, ON Canada; 6 Department of Psychiatry University of Toronto Toronto, ON Canada; 7 Lived Experience Lab Toronto, ON Canada; 8 Department of Psychiatry & Behavioural Neurosciences; School of Nursing Faculty of Health Sciences McMaster University Hamilton, ON Canada; 9 Faculty of Social Science and Humanities University of Ontario Institute of Technology Oshawa, ON Canada; 10 Clinical Public Health Division Dalla Lana School of Public Health University of Toronto Toronto, ON Canada; 11 Division of General Internal Medicine Department of Medicine University of Toronto Toronto, ON Canada; 12 Slaight Family Centre for Youth in Transition Centre for Addiction and Mental Health Toronto, ON Canada; 13 Covenant House Toronto Toronto, ON Canada; 14 Factor-Inwentash Faculty of Social Work University of Toronto Toronto, ON Canada; 15 Faculty of Liberal Arts & Professional Studies York University Toronto, ON Canada; 16 School of Nursing Faculty of Health York University Toronto, ON Canada; 17 School of Medicine Faculty of Education and Health Sciences University of Limerick Limerick Ireland; 18 Applied Health Research Centre Li Ka Shing Knowledge Institute of St Michael’s Hospital Unity Health Toronto Toronto, ON Canada; 19 Division of Biostatistics Dalla Lana School of Public Health University of Toronto Toronto, ON Canada

**Keywords:** youth homelessness, socioeconomic inclusion, transition, critical qualitative methodology, community-based research, identity capital, youth, homeless, rent, community, feasibility, acceptability, novel intervention, socioeconomic, participatory action research, pilot study, protocol, RCT, randomized controlled trial

## Abstract

**Background:**

For young people who have experienced homelessness, relative housing stability alone is insufficient to achieve socioeconomic inclusion. There is little peer-reviewed research investigating interventions targeting socioeconomic inclusion outcomes for youth who have experienced homelessness. Our previous community-engaged work signaled that identity capital (purpose, control, self-efficacy, and self-esteem) may mediate socioeconomic inclusion outcomes for youth exiting homelessness. This 12-month pilot randomized controlled trial (RCT) explores whether portable rent subsidies and an intervention targeting identity capital hold promise as a way to facilitate socioeconomic inclusion for youth exiting homelessness and living in market rent housing in Ontario, Canada.

**Objective:**

The objectives of this study were (1) to examine the feasibility and acceptability of an RCT of targeted economic and identity-based supports to foster socioeconomic inclusion (primary objective), (2) to estimate the effect of adding identity-based supports to economic supports (intervention group) compared with economic supports alone (control group) at the 12-month end point with respect to self-reported proxy indicators of socioeconomic inclusion (secondary objective), and (3) to explore, among the intervention group, whether the estimated effect of the intervention differs by baseline variables or level of engagement with the intervention (exploratory objective).

**Methods:**

This study is a convergent mixed methods, 2-arm parallel RCT, open-label design with 1:1 allocation. All youth participants (n=40) received rent subsidies; half were randomly assigned an identity capital intervention (co-designed leadership guide+coach). The overall study was guided by community-based participatory action research axiology. The qualitative component used a qualitative descriptive design underpinned by critical social theory. The measures used were (1) recruitment, enrolment, and dropout metrics; self-report composite checklists regarding intervention engagement; coaching session attendance; and qualitative focus groups (primary measures); (2) education, employment, and training; housing security; and identity capital (secondary measures); and (3) impact of baseline variables (eg, participant demographics such as gender or mental health symptoms as measured by the Global Appraisal of Individual Needs–Short Screener) or level of engagement with intervention (coaching session attendance) on secondary measures (exploratory measures).

**Results:**

Recruitment and enrolment began March 1, 2023, and ended June 19, 2023. Data collection began March 7, 2023, and ended June 17, 2024. Qualitative and quantitative data analyses concluded on August 20, 2024.

**Conclusions:**

Findings from this RCT will help inform the way we conceptualize the types of supports that are necessary to sustain successful exits from homelessness. The intervention was co-designed with youth who have experienced homelessness, and their voices will continue to inform the next iteration of this work.

**Trial Registration:**

ClinicalTrials.gov NCT05781503; https://clinicaltrials.gov/study/NCT05781503

**International Registered Report Identifier (IRRID):**

DERR1-10.2196/66210

## Introduction

There is a vast literature describing youths’ pathways into homelessness; much less is known about transitions out of homelessness. A common belief is that youth who have experienced homelessness will achieve socioeconomic inclusion—equity in health and well-being [1]—through housing stability. The provision of relatively stable accommodation is seen as a sort of springboard toward socioeconomic inclusion. Longitudinal studies conducted in high-income countries suggest this is often not the case. Despite the attainment of relative housing stability, many young people continue to struggle with day-to-day survival, a lack of purpose, hopelessness, and a sense of being stuck [2-5].

In contrast, there is a dearth of peer-reviewed literature on interventions targeting socioeconomic inclusion outcomes for youth who have experienced homelessness [6]. A 2020 systematic review synthesizing evidence from 53 unique interventions conducted in high-income countries with youth (age 13-25 years) who were experiencing or had experienced homelessness highlights the urgent need for robust evidence on this issue [7]. Most of the reviewed studies focused on interventions related to counseling or treatment (eg, substance use) and many were described as low-rigor designs. Only 42% of the studies involved some form of randomized evaluation; of those, 64% reported mixed, negative, or null findings.

There was only one published study of a randomized intervention offering rent subsidies in the systematic review. This was a secondary analysis of youth (age 18-24 years) in the Canadian study of Housing First in adults (At Home/Chez Soi); there was no systematic adaptation of the intervention for youth. The youth-focused analysis found that Housing First (subsidized housing+intensive mental health supports) was effective for housing stability; however, there were no significant improvements in health and well-being outcomes in the intervention group relative to the control group [8]. The authors of the systematic review concluded, “The field lacks rigorous evaluative evidence of many of the program models on which communities and governments rely to address youth homelessness” [7].

Many of the community collaborators and researchers involved in this study have been working together for several years to try and support successful exits from homelessness and improve socioeconomic inclusion outcomes for youth who have experienced homelessness. In March 2019 (after several months of prestudy collaboration), we began the Transitioning Youth Out of Homelessness (TYOH) study, a 2.5-year pilot community-based mixed method randomized controlled trial (RCT) with youth (mean age 22 years) from 3 cities in the province of Ontario [9]. All participants (n=24) received portable rent subsidies (ie, subsidies not tied to a specific location) for 2 years; 13 were randomly assigned an adult mentor. The overall aim of TYOH (now referred to as TYOH 1.0) was to understand whether youth who received rent subsidies and mentorship achieved better socioeconomic inclusion outcomes relative to the group that only received rent subsidies. We believe TYOH 1.0 was the first published RCT in this population to identify socioeconomic inclusion as the primary outcome (proxy indicators of socioeconomic inclusion encompassed measures such as community integration, self-esteem, and engagement in education, employment, and training).

Quantitative data from TYOH 1.0 revealed that, as a cohort, study participants had stable or nonsignificant improvements in all study outcomes at the primary end point of 18 months compared to baseline (potentially attributable to rent subsidies); however, there were no statistically significant improvements in proxy indicators of socioeconomic inclusion in the mentorship group relative to the nonmentorship group 18 months post randomization [10]. While we were unable to prove that youth receiving rent subsidies and mentorship had significantly better socioeconomic inclusion outcomes compared to the group who received rent subsidies only, there were signals from our quantitative and qualitative data that connecting with informal mentors—people outside the study who played “coach-like” roles (eg, asking powerful and future-oriented questions vs simply providing advice)—was key to fostering socioeconomic inclusion [10].

Drawing on 71 in-depth interviews over the 2.5-year study period, TYOH 1.0 also highlighted the crucial role of identity capital (a sense of purpose, control, self-efficacy, and self-esteem) as an important mediator of socioeconomic inclusion [11]. When people with limited identity capital encounter challenges, they are more likely to give up and take the path of least resistance [12]. Youth in TYOH 1.0 who seemed to more easily navigate their transitions out of homelessness drew on this internal resource of identity capital to push forward despite external challenges [11]. Taking these 2 ideas together (coaching+identity capital) we decided to design a study similar to TYOH 1.0, but with certified coaches (instead of mentors) and a more deliberate targeting of identity capital.

Interventions designed to mediate socioeconomic inclusion by bolstering some or all components of identity capital is an undeveloped area of research but there are some promising transferrable findings from interventions with youth from low socioeconomic backgrounds and with youth who have experienced homelessness [13-18]. In 2018, the principal investigator of TYOH 1.0 and this current study led The Identity Project – a pilot nonrandomized identity capital intervention with youth (n=19) who had experienced homelessness [19]. The results were promising: 9 months after the 6-week 6-session intervention (leadership guide+group coaching) concluded, there were statistically significant improvements and moderate effect sizes in hopelessness and self-esteem compared to baseline [19].

Building on what we learned from our previous work, our team of community and academic partners, including youth with lived expertise, developed TYOH 2.0—a 1-year mixed methods RCT with youth exiting homelessness. The overarching aim was to determine the feasibility and acceptability of a trial focused on identity capital and intended to facilitate socioeconomic inclusion for youth (aged 16-24 years) exiting homelessness and living in market rent housing (refer to Multimedia Appendices 1 and 2 for SPIRIT (Standard Protocol Items: Recommendations for Interventional Trials) and CONSORT (Consolidated Standards of Reporting Trials) checklists). Specifically, the objectives were to (1) examine the feasibility and acceptability of an RCT of targeted economic and identity-based supports to foster socioeconomic inclusion (primary objective), (2) estimate the effect of adding identity-based supports to economic supports (intervention group) compared with economic supports alone (control group) at the 12-month end point with respect to self-reported proxy indicators of socioeconomic inclusion (education, employment and training; housing security; identity capital; secondary objective), and (3) explore, among the intervention group, whether the estimated effect of the intervention differs by baseline variables or level of engagement with the intervention (exploratory objective).

## Methods

### Philosophical Underpinning

This work was grounded in the commitment to centering the voices of youth with lived expertise as well as responding to priorities defined by community partners. The overall study drew on key principles of community-based participatory action research (CBPAR) axiology, and the qualitative component was framed with a critical social theoretical lens [20-23] (Textbox 1).

**Transitioning Youth Out of Homelessness 2.0 philosophical underpinning.**

- Research participants are viewed as experts in their own lives.
- Focus on highlighting how inequitable social structures of power (including the intersection of factors such as race, class, and gender) play out in the lives of participants.
- Search for examples of resilience and agency despite sociostructural inequities.
- A concerted effort to acknowledge and reduce power imbalances between researchers and the community.
- Equal value is placed on academic knowledge and experiential knowledge.
- Commitment to coproducing practical, actionable data to build community capacity and improve the lives of research participants.
- Duty to remain invested in the community beyond the life of the research project.

### Trial Design

This pilot study used a convergent mixed methods (quantitative and qualitative data collected concurrently and the findings combined), 2-arm parallel RCT (participants randomly assigned to either the intervention or control group), open-label (participants and research team aware of random assignment) design with 1:1 allocation (equal number of participants in each study arm) embedded within a CBPAR framework. The study was conducted collaboratively with 4 community partners who work with youth who are experiencing or have experienced homelessness: (1) Covenant House Toronto (Toronto, Ontario), (2) Living Rock (Hamilton, Ontario), (3) The RAFT (St. Catharines, Ontario), and (4) StepStones for Youth (Toronto, Ontario).

The study was prospectively registered on ClinicalTrials.gov (NCT05781503) and received Unity Health Toronto Research Ethics Board approval (#22-230). All protocol amendments were reported to both these organizations. The only protocol deviation was submitted in October 2023, which allowed community partners to send the rent subsidies directly to youth for those at risk of losing their housing due to refusal of third-party payments from landlords, or fear losing their housing due to disclosing a history of homelessness.

### Sample Size

This pilot feasibility and acceptability study was designed with the intention of generating data and hypotheses to inform a full-scale study; therefore, no formal sample size calculation was performed. Instead, we chose a pragmatic sample size of 40 participants, based on the financial resources available to provide rent subsidies and coaching over a 12-month period.

### Participants

Eligible youth ages 16-24 years who had left homelessness (defined by the Canadian Observatory on Homelessness [24] as unsheltered, emergency sheltered, provisionally accommodated, and at risk of homelessness) within the past 12 months and living or planning to live in market rent housing were identified by our community partners. This age mandate was chosen because this is the age group served by our community partners. We chose to target the first year of exiting homelessness because our collective experience has shown that this is a particularly precarious time for youth in terms of mental health challenges and the risk of returning to homelessness. Foster care was included in our definition of homelessness given the time-limited and provisionally accommodated nature of these living arrangements, which put youth at high risk of homelessness.

In addition to the above conditions, recruited and enrolled participants were required to meet the following criteria (Textbox 2).

**Inclusion and exclusion criteria.**

**Inclusion criteria**

- Were able to provide free and informed consent.
- Were able to understand English (intervention and data collection conducted in English).
- Were willing to actively participate in the intervention (co-designed leadership guide+coach) if randomized to this arm.

**Exclusion criteria**

- In imminent danger of losing their housing and not being able to use the rent subsidy to sustain market rent housing (eg, facing jail time).
- Already receiving rent subsidies (eg, Canada-Ontario Housing Benefit).
- Enrolled in a program or study with similar features to the Transitioning Youth Out of Homelessness 2.0 intervention (eg, individual coaching or program targeting identity capital).

### Identification and Consent

Study participants were recruited from the cities in which our community partners are located: Toronto, Ontario (Greater Toronto Area population 6.7 million), Hamilton, Ontario (population 785,000), and St. Catharines, Ontario (St. Catharines-Niagara population 416,000). The initial introduction to the study was facilitated by our community partners. Frontline staff (eg, case workers, housing workers) working with youth who have experienced homelessness were responsible for identifying youth who may be interested in the study and met the inclusion and exclusion criteria. Staff provided potential participants with information about the study and the study consent form to review. Potential participants were directed to contact the study lead research coordinator by email or phone, who then conducted eligibility screening over the phone. Once participants were deemed eligible for the study, they were connected to a member of the research team. To build rapport with community partners and study participants, one dedicated research team member was assigned to each partner site.

Each research team member reached out to eligible youth for an initial Zoom call. Free and informed verbal consent was first obtained after carefully reviewing the information and consent form with the participant. During the same Zoom call, electronic consent was also obtained once the research team member emailed participants the secure baseline data collection web link and a unique login and password. The baseline data collection questionnaire was programmed so that participants were unable to proceed until they selected “Yes” for consent. The research team member stayed on the call while the participants completed the baseline questionnaire.

### Randomization and Allocation

**Figure 1:**
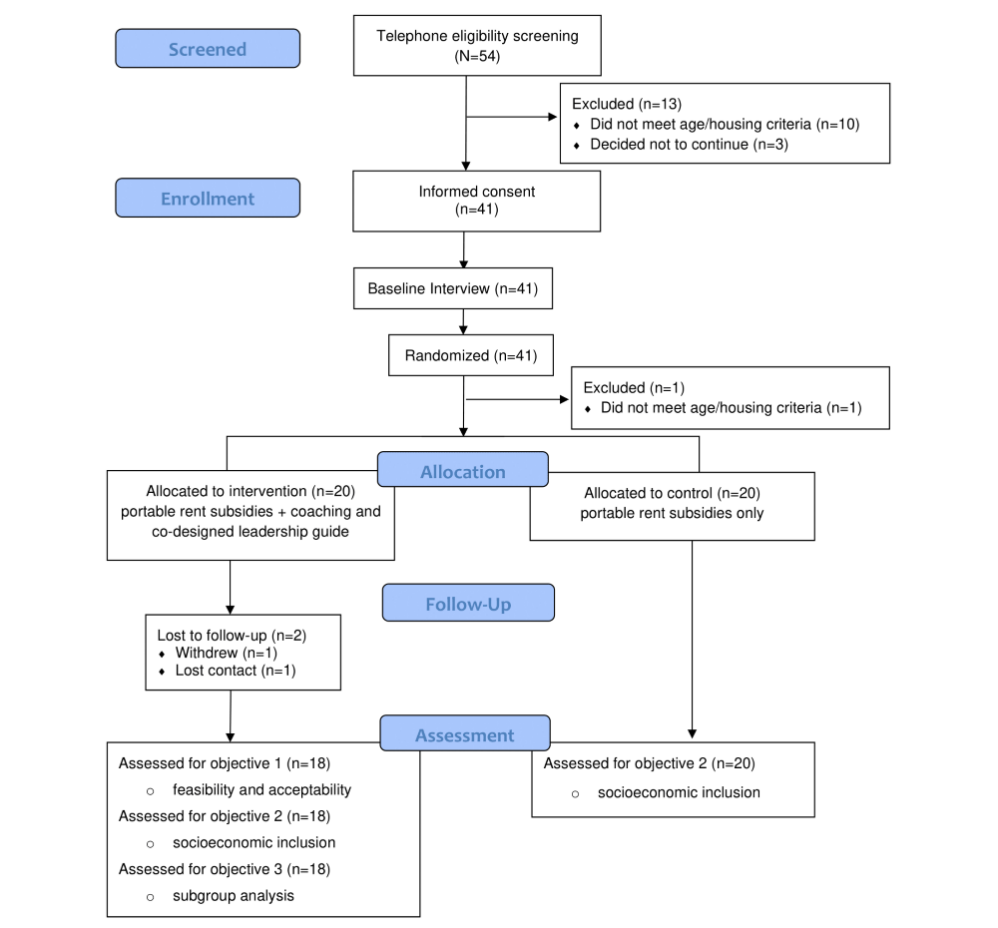
CONSORT diagram for pilot and feasibility studies of the anticipated flow of participants through the study.

Once participants completed the baseline questionnaire the research team member opened a sequentially ordered randomization file from a sequentially ordered electronic randomization folder to obtain the participant’s group assignment. The participant’s group allocation was noted, and participants were informed immediately if they had been allocated to the intervention (rent subsidies and co-designed leadership guide+coach) or control (rent subsidies alone) group (Figure 1 [25]). Randomization was stratified by site using block randomization (random block sizes of 2 and 4). In keeping with typical community-based RCTs with psychosocial interventions, blinding of participants, community partners, and research staff to allocation group was not pragmatic due to the nature of the intervention [26].

Further to Figure 1, the participant excluded post randomization was found to be ineligible before the intervention was initiated. We will not be using data from the ineligible participant. A new youth was then enrolled and randomized. In addition, the participant who withdrew during follow-up did so in order to meet the criteria for a different (longer-term) rent subsidy program.

### Intervention

Youth in both arms (n=40) were provided monthly rent subsidies (CAD $700/month Hamilton and St. Catharines; CAD $800/month Toronto) for 12 months, which was generally paid directly to landlords and facilitated by our community partners (refer to Trial Design re: protocol deviation; at the time of recruitment, the conversion rate was CAD $1=US $0.7357). Youth who participated in TYOH 1.0 suggested the TYOH 2.0 research team should encourage participants to delegate a portion of the money they would have spent on rent toward a savings account, which they would be able to access at the end of the study. This idea received unanimous support from our community partners and was suggested to all participants at enrolment and suggested periodically during the study. Youth randomized to the intervention group (n=20) were also provided a co-designed leadership guide and assigned a study coach. The control group (n=20) was offered the co-designed leadership guide at the end of the study.

### Co-Designed Leadership Guide

The strengths-based leadership guide (Finding Home: A Guide for Youth in Transition) was co-designed with 12 youth who had experienced homelessness, including youth who participated in TYOH 1.0. NST drafted the original version of the guide and cofacilitated a full-day co-design workshop with research team member MD. MD is an experienced peer engagement specialist who draws on her lived expertise of homelessness to collaborate with researchers and youth who have experienced homelessness. Significant enhancements were made to the guide based on feedback from workshop participants.

The Leadership Guide contained 12 chapters with the overarching aim of enhancing identity capital along with providing strategies to achieve participant-identified goals (Table 1). Each chapter contained 4 activities (eg, a self-reflection exercise or listening to a podcast). It was suggested youth complete one chapter every month; however, they were also encouraged to go at their own pace. Copies were provided in print and electronic formats. While the guide was designed for individual study, it was also meant to serve as a reference point for individual and group discussions with the study coaches.

**Table 1:** Co-designed leadership guide.

Chapter	Key learning
1. The “Mind Hack”	Learning how to regulate the brain through mindfulness training
2. Living Core Values	Exploring the link between core values and a sense of mastery
3. Leading with Your Mind	Understanding the connection between core values, activities, and mood
4. Check-In	Assessing progress and considering what strategies need refinement
5. Building A Good System	Developing successful strategies to live out core values
6. Identity-Based Habits	Examining the link between identity and habits
7. Simple Strategies to Get Stuff Done	Exploring simple techniques to boost efficiency
8. Check-In	Assessing progress and considering what strategies need refinement
9. The Role of Grit	Examining the relationship between mindset and grit (passion and perseverance)
10. Wholehearted Living	Learning to connect with and belong to oneself
11. Leading with Courage and Purpose	Overcoming shame and cultivating self-worth
12. Check-In	Assessing progress and considering what strategies need refinement

### Study Coaches

The role of the coach was to speak with youth about how to draw on internal resources to help orient them toward their preferred future. This role was different from case management or mentorship in that a specific coaching methodology (Brief Solution-Focused Coaching) [27] was used to walk alongside youth as they developed strategies to reach self-defined goals (Table 2). Each coach was instructed to devote 2.5 days/week to the intervention and was given a caseload of 9-11 youth. The coaches were instructed to meet individually with each youth on their caseload every two weeks and collectively with the rest of the youth in their group every month.

**Table 2:** Coach training^a^.

Training	Focus	Method
Foundations of Brief Coaching	Solution-Focused theory and Brief Coaching framework (solution-building vs. problem-solving)	Asynchronous online modules (approximately 24 hours in length)
Applications of Brief Coaching	Strategies to co-construct a preferred future with clients	Asynchronous customized virtual training (approximately 24 hours in length)
Coaching masterclass case conferences	Coaching dialogue analysis Providing and receiving peer feedback	90-minute live virtual sessions (every two – four weeks for 12 months)
Supervision and mentorship	Opportunity for coaches to connect with coach mentor, Dr. Haesun Moon	Available throughout the 12-month study Designated check-ins at 6 and 9 months

^a^Coach training conducted by the Canadian Centre for Brief Coaching [27].

The 2 study coaches were selected by a panel that included 2 leaders from our community partner organizations, 2 research team members, and 3 youth from TYOH 1.0 who work as paid experts on the TYOH 2.0 Community Partner and Researcher Advisory Board. Each of the coaches had their own coaching practices outside the homelessness sector, which they maintained during the study. The coaches met monthly with our youth advisors and team members to provide updates as to how the coaching sessions were progressing.

### Study Outcomes

**Figure 2:**
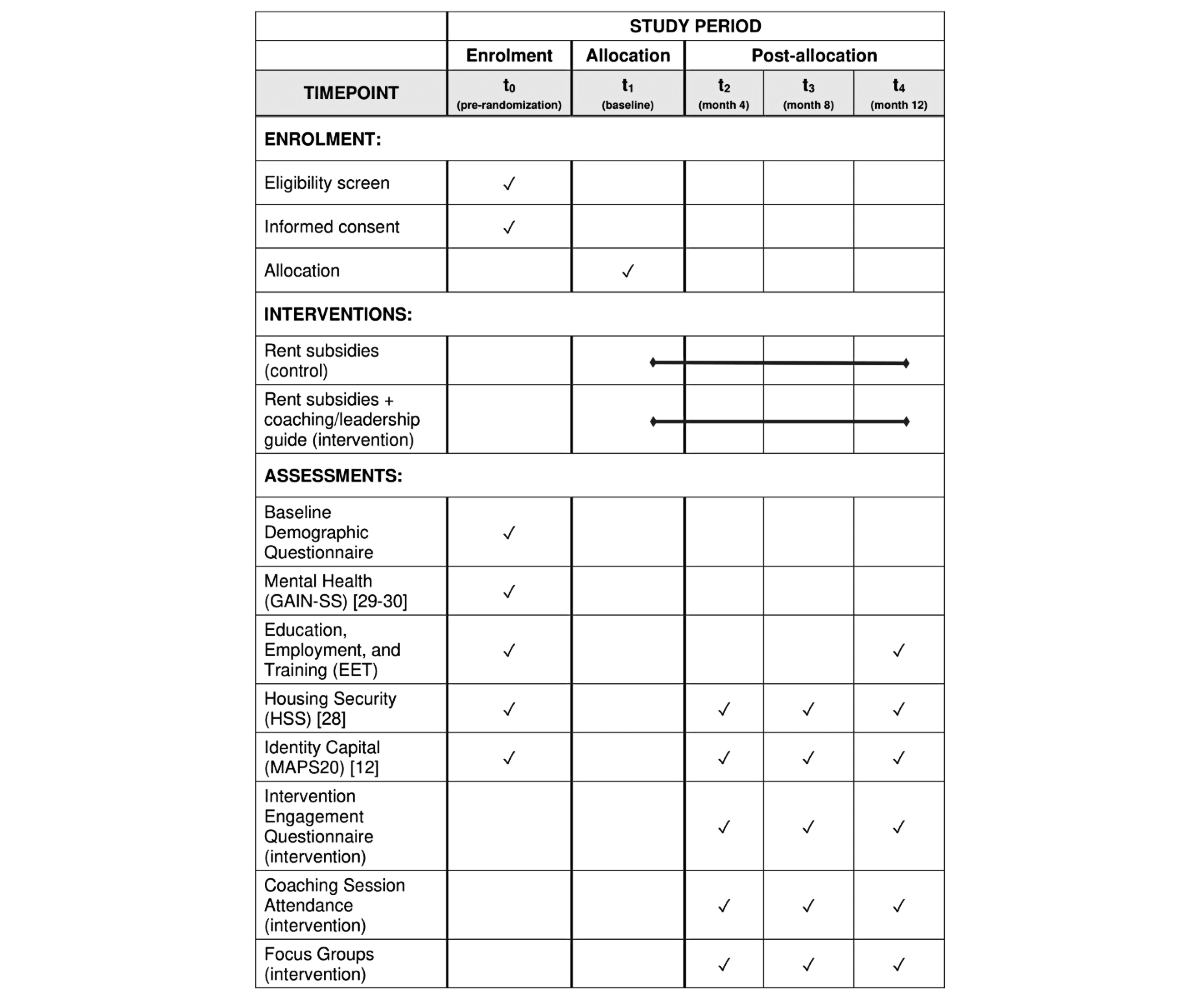
SPIRIT schedule of enrolment, interventions, and assessments [31].

The primary outcomes of this pilot study were feasibility and acceptability (Table 3). Secondary outcomes included proxy indicators of socioeconomic inclusion based on our clinical expertise and research with this population: (1) education, employment, and training; (2) housing security; and (3) identity capital (Table 3). The relative timing of interventions and assessments is described in Figure 2.

**Table 3:** Key outcome variables, domains, and instruments

Key outcome variables and domains			Instruments
**Primary outcomes**			
	Feasibility and acceptability	Recruitment and enrollment metrics Coaching session attendance (recorded by coaches) Intervention engagement questionnaire (includes items on engaging with leadership guide and frequency of contact with coach) Dropout metrics Focus groups	
**Secondary outcomes and domains** **(proxy indicators of socioeconomic inclusion)**			
	Education, employment, and training	Composite education, employment, and training checklist (includes items on current school, work, and training or trades programs)	
	Housing security	Housing Security Scale–V.3 [29] (subscales: housing need, subjective stability, safety net, threats to stability)	
	Identity capital	Multi-Measure Agentic Personal Scale [12] (subscales: self-esteem, purpose in life, internal locus of control, self-efficacy)	
**Exploratory domains**			
	Demographics	Baseline demographics questionnaire (includes age, gender, race or ethnicity, sexual orientation, immigration, child welfare, homelessness history, and formal and informal supports)	
	Baseline mental health	Global Assessment of Individual Needs-Short Screener V.3.0.2 [30,31] (subscales: internalizing disorder, externalizing disorder, substance disorder, crime, and violence)	

### Decision to Scale

The decision to proceed to an adequately powered definitive trial will be based on feasibility and acceptability (Table 3). The research team is particularly interested in quantitative metrics pertaining to coaching session attendance (eg, number attended out of a possible 24 sessions), leadership guide engagement, and intervention dropout rates, as well as qualitative data from the focus groups. We predetermined that we would not proceed to a definitive trial if we found intervention attendance or engagement was less than 50% (ie, average coaching attendance of less than 12 sessions over one year), more than 30% of participants (n=6) dropped out of the intervention, or qualitative feedback from intervention participants during focus group sessions was overwhelmingly negative.

### Quantitative Methods

Questionnaires were completed via a secure weblink, which was emailed to participants along with a unique login and password, at baseline and 4-, 8-, and 12-months post randomization. Those unable to access the weblink were offered the option of using computers at MAP Centre for Urban Health Solutions or at their respective community partner agency.

### Quantitative Analysis

Quantitative analysis was performed using the intention-to-treat principle; that is, all participants were included and analyzed in the groups they were originally randomized. Baseline characteristics of the intervention and control groups were summarized using descriptive statistics (ie, mean, SD, median, and IQR for continuous variables, and frequencies and proportions for categorical variables).

#### Primary Outcomes

Primary outcomes were analyzed by estimating the recruitment rate as the proportion of contacted individuals who expressed interest in participating in the study. The enrollment rate was calculated as the proportion of recruited individuals who were eligible and consented to participate in the study. Dropout rates were separately calculated for intervention and control groups at the end of the study as 1 – proportion of randomized participants who completed the study at 12 months. Exact (Clopper-Pearson) 95% confidence limits were also calculated. Among those in the intervention group, acceptability was measured as the percentage of coaching sessions attended over the 12-month period, out of a maximum of 24.

#### Secondary Outcomes

Secondary outcomes were analyzed by calculating descriptive statistics at each study time point and exploring differences in trajectories from baseline to 12 months follow-up between intervention and control groups using scatterplots and box plots. Adjusted mean group differences with 95% CI in continuous outcomes at 12 months (housing security and identity capital) between participants who received the intervention and control participants were estimated using analysis of covariance (ie, linear regression models), including an indicator of the intervention group and the baseline value of the outcome, adjusting for the site. We performed regression diagnostics and repeated analyses using the nonparametric Wilcoxon rank-sum test if there were extreme outliers or influential observations.

The education, employment, and training (EET) indicator was a composite binary outcome for education employment or training based on the EET questionnaire. EET was 1 if “Attending secondary (eg, high school) or postsecondary (eg, college or university) classes,” “Employed in the past month,” or “Participating in a paid or unpaid training or trades program.” Otherwise, EET was 0. We compared groups with respect to the housing need indicator and EET at 12 months by estimating differences in proportions. A logistic regression model estimated odds ratios with 95% CI, adjusted for site.

#### Exploratory Subgroup Analysis

Exploratory subgroup analysis was conducted considering only the intervention group and stratifying the primary outcomes by selected baseline demographics (for example, gender) or severity levels of components of the Global Assessment Individual Needs-Short Screener [30,31]. Severity levels are: 0 problems=low (unlikely to have a diagnosis or need services); 1-2 problems=moderate (a possible diagnosis; the client is likely to benefit from a brief assessment); 3 or more problems=high (high probabilities of a diagnosis; the client is likely to need more formal assessment and intervention, either directly or through referral). We also calculated the correlation between secondary outcomes and the level of engagement with the intervention measured as the percentage of class sessions attended out of a maximum of 24 over the 12-month period. Spearman correlation and Spearman partial correlation coefficients were calculated.

### Interim Analysis

There was no interim quantitative analysis planned to guide a decision to stop the study early for this pilot study [25]. We decided before the study began to either stop the intervention early or make adjustments to the intervention if feedback from participants during the focus group sessions was overwhelmingly negative and/or the majority of youth stopped participating in the intervention.

### Qualitative Methods

Focus groups with those in the intervention group were conducted by NST and MD in Toronto, Hamilton, and St. Catharines at 4, 8, and 12 months post randomization. Focus group questions primarily centered around intervention acceptability but also explored the impact of the intervention on identity capital and socioeconomic inclusion (eg, connection to broader social networks). Analysis began during and after the first data generation session, meaning the questions asked evolved over time based on our preliminary interpretations of the data [32-34]. Keeping in mind that data generation context impacts participant responses [34-36], focus groups were held in a community setting to help minimize researcher-participant power imbalance and increase participant comfort. Participants unable to attend in-person focus groups were offered the option of virtual attendance.

All focus groups were audio recorded using a password-protected device. Audio recordings were transcribed verbatim by a member of the research team, and the transcripts were uploaded to the web-based application Dedoose [37] for storage and retrieval. One member of the research team served as an observer and note taker at each focus group session to document nonverbal communication (eg, eagerness or disinterest) as well as preliminary analytic insights based on listening to the discussion. In addition, each focus group facilitator documented field notes as soon as possible after the meeting to capture their own observations and reflections on the sessions [33-35]. Food was also provided at the focus groups.

### Qualitative Analysis

The team used reflexive thematic analysis to help make sense of the data [38]. Reflexive thematic analysis is comprised of 6 iterative phases (data familiarization, coding, initial theme generation [patterns anchored by a shared idea or concept], theme development and review, theme refining, and writing up). Crucially, this form of analysis also requires engagement with theory (eg, critical social theory), reflexivity (awareness of social location on analysis), and interpretation (findings not seen as self-evident) [38]. Before each monthly qualitative data analysis session, 2 team members read the most recent focus group transcript multiple times, coded (“tagged”) data relevant to intervention acceptability and our assumption that identity capital was a mediating factor in socioeconomic inclusion (and looked for data that might disprove that assumption), and compared codes to previous transcripts [32,33,38].

At the monthly analysis sessions led by NST, codes were discussed (and revised or deleted as needed) and organized in a code book, clustered into initial themes, and eventually refined and synthesized into key themes. Analysis was primarily inductive (moving from data to conceptualizing); however, deductive reasoning (moving from conceptualizing to data) was used when we wanted to understand new data through the lens of our emerging conceptual framework. The analysis team also formulated new focus group questions based on our preliminary analytic insights. Focus group participants were asked for their perspectives on the emerging interpretations during subsequent focus groups, and these perspectives played a key role in helping shape the data analysis.

As in TYOH 1.0, the aim of our analysis was to go beyond superficial understandings of intervention acceptability. Instead, we engaged in “value-adding” qualitative analysis—interpreting, contextualizing, and adopting a critical posture (eg, attention to power)—with the intention of operationalizing key conceptual insights in a conceptual framework [39]. As we did this, our team embraced the “creative presence of the researcher,” drawing on our own knowledge, insights, and experiences (including the experience of homelessness) to help make sense of the data [39].

### Participant and Public Involvement

A genuine partnership with the community—including youth with lived expertise—has been woven into this study from inception to dissemination. As noted previously, we have been working with most of the community partners on this study for several years, and the leadership guide being used in the intervention was co-designed with youth who have experienced homelessness. Three participants from TYOH 1.0 worked as paid advisors on the TYOH 2.0 Community Partner and Researcher Advisory Board. The Board met approximately every 3 months from June 2022 to October 2024. Our community partners and youth advisors were actively involved in planning TYOH 2.0, including discussions around research questions, outcome measures, recruitment, the burden of the intervention, and the time required to participate.

### Ethical Considerations

We endeavored to weave ethical considerations into all aspects of the study design, including our decision to use a CBPAR methodology, ensure all participants received portable rent subsidies (not just the intervention group), and offer the co-designed leadership guide to those in the control group after the study was over. The consent form highlighted that participants could withdraw from the entire study or just the identity capital intervention at any time, and this would not impact their monthly rent subsidy. Participants received honoraria via e-transfer within 24 hours of each data collection session (CAD $20 [US $14.28] for completing quantitative questionnaires and CAD $40 [US $28.57] for attending focus groups). Efforts were made to mitigate potential privacy and confidentiality concerns at all stages of the study (Table 4).

**Table 4:** Privacy and confidentiality.

Potential concerns	Mitigation strategies
Recruitment	Interested participants reached out to the study lead research coordinator rather than community partners sharing contact information with the team.
Eligibility screening	No personal health information or personal identifying information was collected.
Enrollment	There were no signed paper consent forms; instead, electronic consent (after verbal consent) was required before baseline data collection. The consent form indicated that limits to confidentiality applied if a participant disclosed that they intended to hurt themselves or others, or if they informed a member of the research team that someone under the age of 16 years was experiencing abuse or neglect.
Quantitative data collection	The MAP SRU^a^ programmed the questionnaires into Snap Professional software [40] and provided a secure weblink along with unique logins and passwords for data collection. The electronic data was kept on the Snap server that is owned and operated by the SRU and is located inside Unity Health Toronto’s secure network at SMH^b^. Deidentified data was downloaded directly to the SMH secure server.
Qualitative data collection	Focus groups were audio recorded on a password-protected application on a password-protected device. The audio-recorded files were securely sent to a research team member via Unity Health email with a link that expired in 24 hours. After the team members had transcribed the audio file, they deleted the file. We had a contingency plan that if focus groups had to pivot to virtual because of pandemic-related concerns, we would use a Zoom platform, enable end-to-end encryption, and only record the audio (not video). All transcripts were stored on an SMH secure server and uploaded to Dedoose [37]. Pseudonyms (chosen by participants) were used in focus group transcripts. All notes pertaining to the focus groups (eg, participant observation and field notes) were stored on an encrypted USB key and the files were transferred to the SMH secure server as soon as possible after each focus group.
Linking log	A key that linked each participant’s name with their participant identification number and pseudonym was created by the lead research coordinator and stored as a separate electronic file on the SMH secure server.
Data access	Only authorized members of the research team have access to quantitative and qualitative study data, and an access log was maintained by the lead research coordinator. Deidentified raw data will be made available upon reasonable request (eg, request comes from a researcher affiliated with an academic institution).
Data retention	All data will be destroyed after 10 years. NST will be responsible for ensuring the data is destroyed.

^a^SRU: Survey Research Unit.

^b^SMH: St. Michael’s Hospital.

We intentionally did not choose deficit-focused questions or scales for the T2-T4 quantitative data collection sessions, and questionnaires were programmed to allow participants to skip questions if they chose. The focus group questions also targeted participant strengths, and participants were reminded at the start of each session that they did not have to answer any questions that made them uncomfortable. NST and members of the research team met monthly with the study coaches. These meetings provided an opportunity to assess or discuss any unintentional harms or risks to intervention participants, which were followed up immediately.

The study was approved by the Unity Health Toronto Research Ethics Board (REB# 22-230). Any protocol amendments were approved by the research ethics board. Free and informed verbal and electronic consent was obtained from all participants before the initiation of intervention and research activities.

## Results

Recruitment and enrolment began March 1, 2023, and ended June 19, 2023. Data collection began March 7, 2023, and ended June 17, 2024. Qualitative and quantitative data analyses concluded on August 20, 2024.

## Discussion

### Anticipated Outcomes

We anticipate that this pilot study will provide important new insights regarding the feasibility and acceptability of an intervention using coaching and a co-designed leadership guide to target identity capital for youth transitioning out of homelessness. Although the study is not powered to detect statistically significant differences in the proxy indicators of socioeconomic inclusion outcomes, we hope to see signals in this data—especially in the Multi-Measure Agentic Personal Scale [12]—that the intervention holds promise. As we have found with our previous research [10,11], we anticipate the rigorous qualitative component will strongly influence our decision-making as to the next steps.

Homelessness in Canada is increasing, with 44% of those experiencing homelessness reporting they were first homeless before the age of 25 (with a spike between the ages of 15-19 years of age) [41]. This represents a crucial missed opportunity for homelessness prevention. Interventions like TYOH 2.0 offer promise as a way to prevent young people from returning to homelessness and potentially becoming chronically homeless adults. To be clear: we are not suggesting with this intervention that young people should “bootstrap” themselves out of homelessness. Rather, our aim is to provide young people with some of the tangible (eg, money toward rent) and intangible (eg, fostering identity capital) resources typically provided or nurtured—at least in part—through living at home.

Over 60% of Canadian youth (20-24 years of age) live with at least one parent [42]. This is important to consider when thinking about what to expect from young people attempting to transition out of homelessness. At a minimum, we need to adopt a holistic response to youth homelessness and think beyond housing when considering what supports might foster socioeconomic inclusion. We anticipate using the findings from this study to develop a multicomponent intervention that incorporates aspects of this current intervention (potentially revised) along with components that may need to be added to better facilitate socioeconomic inclusion for youth exiting homelessness.

### Dissemination Plan

Working alongside community partners to disseminate findings with the aim of highlighting sociostructural inequities, building community capacity, and improving the lives of the youth we work alongside is fundamental to this work. We anticipate disseminating our findings broadly in a variety of formats such as oral presentations, open-access plain language summaries or reports, and scientific journal papers. We are committed to dissemination beyond academic audiences. For example, for TYOH 1.0, we produced an animation of our study findings along with a documentary film [43]. Whenever possible, we intend to discuss our study findings alongside a lived expert involved in this research.

### Limitations

This study has limitations. All youth were connected to urban-based social service agencies in the province of Ontario; youth living in rural locations and outside of Ontario may not take up the intervention the same way. In addition, all of the quantitative instruments were based on self-reports and thus subject to social desirability bias. We also chose quantitative measures that we believe signal socioeconomic inclusion; it is plausible that these measures do not adequately capture this complex concept. Finally, this pilot study was not powered to detect statistical significance; findings beyond feasibility and acceptability must be interpreted with caution.

### Conclusions

Findings from this RCT will help inform the way we conceptualize the types of support that are necessary to sustain successful exits from homelessness. This is important given the limited peer-reviewed evidence base on interventions designed to foster socioeconomic inclusion for youth exiting homelessness. The intervention was co-designed with youth who have experienced homelessness, and their voices will continue to inform the next iteration of this work.

## Appendix

Multimedia Appendix 1 Completed SPIRIT 2013 checklist: Recommended items to address in a clinical trial protocol and related documents.

Multimedia Appendix 2 Completed CONSORT 2010 checklist of information to include when reporting a pilot or feasibility trial.

## Abbreviations

CBPAR: community-based participatory action research
CONSORT: Consolidated Standards of Reporting Trials
EET: education, employment, and training
RCT: randomized controlled trial
SPIRIT: Standard Protocol Items: Recommendations for Interventional Trials
TYOH: Transitioning Youth Out of Homelessness

## Data Availability

The datasets generated or analyzed during this study are available from the corresponding author on reasonable request.
